# C-reactive protein is an independent predictor for carotid artery intima-media thickness progression in asymptomatic younger adults (from the Bogalusa Heart Study)

**DOI:** 10.1186/1471-2261-11-78

**Published:** 2011-12-30

**Authors:** Ahmet Toprak, Ramprasad Kandavar, Demet Toprak, Wei Chen, Sathanur Srinivasan, Ji Hua Xu, Asif Anwar, Gerald S Berenson

**Affiliations:** 1 Tulane University School of Medicine, Heart and Vascular Institute, New Orleans, LA, USA; 2 Tulane University School of Medicine, Department of Internal Medicine, New Orleans, LA, USA; 3 Tulane University School of Medicine, Department of Pediatrics, New Orleans, LA, USA; 4 Tulane Center for Cardiovascular Health, New Orleans, LA, USA

**Keywords:** Carotid artery intima-media thickness progression, cardiovascular risk, c-reactive protein, epidemiology, young adults

## Abstract

**Background:**

Conflicting information exists regarding the association between hsCRP and the progression of early stages of atherosclerosis. The purpose of the study was to investigate the association of high sensitiviy c-reactive protein (hsCRP) along with major cardiovascular (CV) risk factors on early carotid atherosclerosis progression in a large, population-based cohort study.

**Methods:**

The study cohort included 839 young adults (aged 24 to 43 years, 70% white, 42% men) enrolled in Bogalusa Heart Study, who in 2001-2002 attended baseline examination with measurements of CV risk factors. Progression of carotid artery intima-media thickness (IMT) was assessed during a mean follow-up of 2.4 years.

**Results:**

Carotid artery IMT progression rates were as follows: composite carotid artery = 9.2 ± 52 μm/y, common carotid artery = 0.0 ± 51 μm/y, carotid bulb = 8.8 ± 103 μm/y, and internal carotid artery = 18.9 ± 81 μm/y. Elevated baseline hsCRP, reflecting an inflammatory state, showed independent association with composite carotid artery IMT progression. Increased age, systolic blood pressure, fasting glucose, LDL cholesterol, and current smoking were other risk associates of carotid artery IMT progression in young adults, indicating an underlying burden on the CV system by multiple risk factors.

**Conclusion:**

In this population-based study, we observed independent categorical association of increased hsCRP with carotid artery IMT progression in young adults. This study underlines the importance of assesssing hsCRP levels along with smoking and traditional CV risk factor profiles in asymptomatic young adults.

## 1. Background

Carotid artery intima-media thickness (IMT), as assessed noninvasively by ultrasonography, is a useful measure of preclinical atherosclerosis and has been found to predict future risk for myocardial infarction, stroke, and death from coronary heart disease. A change in carotid artery IMT over time has been validated as a vascular marker of the progression of atherosclerosis [1]. Such longitudinal changes in carotid artery IMT provides further pathophysiologic insights into atherogenesis. Childhood risk factors were associated with carotid artery IMT in young adulthood in the Bogalusa Heart Study [2], and an increasing risk factor burden was associated with increased aortic and coronary atherosclerosis found at autopsy in young adults [3]. Although traditional CV risk factors for atherosclerosis are well known [4,5], increasing evidence implicates inflammation in the pathogenesis of atherosclerosis and CV disease. Independent association between elevated high sensitivity c-reactive protein (hsCRP) and carotid artery IMT progression has revealed contradictory results [6-12]. High sensitivity CRP may reflect an exaggerated inflammatory response associated with traditional CV risk factors or reflect the causal role of inflammation in the initiation and/or progression of atherosclerosis, further observations are needed.

As part of the Bogalusa Heart Study, a biracial (black-white) community based epidemiological study of the early natural history of cardiovascular (CV) disease, the present study examines the relationships of baseline traditional CV risk factors and hsCRP to carotid artery IMT progression in asymptomatic younger adults by race and sex.

## 2. Methods

### 2.1.Study cohort and design

The Bogalusa Heart Study is a long-term epidemiologic study of the early natural history of arteriosclerosis in children and young adults from a semirural, biethnic (65% white, 35% black) community in Bogalusa, Louisiana [13]. This report includes a cohort of 839 subjects (70% white, 42% men) who were examined in both the 2001-2002 survey (mean age, 36.6 ± 4.3 years; range, 24 to 43 years) and the 2003-2005 survey (mean age, 39.0 ± 4.3 years; range, 26 to 46 years) for CV risk factors and carotid artery IMT. All subjects in this study gave informed consent for examinations. Study protocols were approved by the Institutional Review Board of the Tulane University Health Sciences Center.

### 2.2. General examinations

Standardized protocols were used by trained examiners [14]. Height and weight were measured in triplicate, and the mean values were used to calculate body mass index (BMI = weight in kilograms divided by the square of the height in meters) as a measure of overall obesity. Mean waist circumference divided by height was used as an indicator of visceral fatness. Right arm blood pressure was measured in triplicate with mercury sphygmomanometers by 2 trained observers while subjects were seated and relaxed. The first and fifth Korotkoff phases were used to determine systolic and diastolic blood pressure, respectively; means of 6 replicate readings were used.

### 2.3. Laboratory analysis

Subjects were instructed to fast for 12 hours before screening, and compliance was determined by interview on the morning of examination. Plasma glucose was measured by a glucose oxidase method as part of the multiple chemistry profile (SMA20) in the multichannel Olympus Au-5000 Analyzer (Olympus, Lake Success, NY). A radioimmunoassay kit was used to measure plasma insulin (Phadebas insulin kit, Pharmacia Diagnostics, Piscataway, NJ). Serum cholesterol and triglycerides were determined enzymatically on the Hitachi 902 Automatic Analyzer (Roche Diagnostics, Indianapolis, Ind.). Serum lipoprotein cholesterols were analyzed by a combination of heparin-calcium precipitation and agar-agarose gel electrophoresis procedures. Plasma high sensitivity c-reactive protein (CRP) was measured by latex particle-enhanced immunoturbidimetric assay on the Hitachi 902 Automatic Analyzer. Urinary albumin excretion was assessed on a morning spot urine sample using an enzyme-linked immunosorbent assay kit (Exocell, Philadelphia, Pa.). The laboratory is being monitored by a surveillance program of the Centers for Disease Control and Prevention, Atlanta, Ga. Intraclass correlation coefficients, a measure of reproducibility of the entire process from blood collection to data processing, between the blind duplicate values (n = 103) were 0.98 for total cholesterol, 0.99 for high-density lipoprotein (HDL) cholesterol, and 0.98 for glucose.

### 2.4. Carotid ultrasonography

Trained sonographers performed ultrasound examinations using the Toshiba Ultrasound instruments (Toshiba SonoLayer SSH 160A and Toshiba Power Vision SSH-380, Toshiba America Medical Systems, Tustin, Ca.) with 7.5-MHz linear array transducers. B-mode images were recorded at the common catotid, carotid bulb (bifurcation), and internal carotid arteries bilaterally according to previously developed protocols for the Atherosclerosis Risk in Communities study [15]. Images were recorded on S-VHS 1/2" tapes and read centrally by certified readers at the Division of Vascular Ultrasound Research, Wake Forest University School of Medicine, using a semiautomatic ultrasound image processing program developed by the California Institute of Technology Jet Propulsion Laboratory (Pasedena, Ca.) according to strict protocols [16]. Previous readings from 2001-2002 were accepted as baseline values. The mean of the maximum carotid IMT readings of 3 right and 3 left far walls for common, bulb, and internal segments was used. Images were read at the same cardiac cycle, which was end diastole. If bilateral images were not available, the value of one side was used as the mean. The trained sonographers were blinded to risk factor data. Reproducibility (mean difference ± SD) of composite carotid artery IMT (average of the segmental maximum carotid IMT measurements) in 141 subjects (69 from 2001-2002 survey and 72 from 2003-2005 survey) was 15.1 ± 84.4 μm for mean maximum thickness corresponding with a 99% confidence interval (CI) of -3.4 to 33.7 μm.

### 2.5. Statistical methods

All data analyses were performed using SPSS software (SPSS Inc., Chicago, Il.). Insulin, triglycerides and urinary albumin-creatinine ratio were log-transformed to improve the normality of distribution; however, their mean values in original scales are presented for description. Differences in mean values of study variables between race-sex groups were tested by analysis of covariance models. Cardiovascular risk factors and carotid artery IMT at each survey were described by mean ± SD values and contrasted by paired t-test or McNemar test. The impact of the correlates on composite carotid artery IMT progression was examined by stepwise linear regression model in the total sample. Composite carotid artery IMT progression was adjusted for the baseline (2001-2002 survey) composite carotid artery IMT and the risk model included baseline age, gender, race, systolic and diastolic blood pressures, waist-height ratio, fasting glucose, serum low-density lipoprotein (LDL) cholesterol, HDL cholesterol, fasting triglycerides, high-sensitivity c-reactive protein ≥ 2 mg/l and smoking status with a p-value criteria of < 0.05.

## 3. Results

Table 1 shows the baseline and follow-up CV risk factors at each survey by race and sex. Significant race differences were noted in both sexes for systolic and diastolic blood pressures (whites < blacks), and the prevalence of high sensitivity c-reactive protein ≥ 2 mg/l (whites < blacks); however, the race differences in age (whites > blacks), BMI (whites < blacks), waist-height ratio (whites < blacks), glucose (whites < blacks), insulin (whites < blacks), and triglycerides (whites > blacks) were significant only in females. The race difference in HDL cholesterol (whites < blacks), total cholesterol-HDL cholesterol ratio (whites > blacks), and the prevalence of current smoking (whites < blacks) were significant only in males. Compared with women, men showed higher values of systolic and diastolic blood pressures, serum triglycerides, and total cholesterol-HDL cholesterol ratio; men had lower BMI and higher prevalence of current smoking in blacks only; however, men had lower HDL cholesterol and the prevalence of high sensitivity c-reactive protein ≥ 2 mg/l in whites only. Waist-height ratio and serum insulin were higher white men > white women and black women > black men. Significant increases in age (all race-sex groups), BMI (whites), waist-height ratio (white women), serum glucose (all except black men), LDL cholesterol (white men), and HDL cholesterol (white women); however significant decreses in diastolic blood pressure and total cholesterol-HDL cholesterol ratio (black men) were observed between 2 surveys. Significant sex difference for BMI and waist-height ratio were no longer evident in the follow-up survey, while glucose showed a significant sex difference (white men > white women).

**Table 1:** Baseline and follow-up cardiovascular risk characteristics of study cohort by race and sex

	White		Black		P value*	
	
Baseline survey (2001-2002)	Men (n = 266)	Women (n = 324)	Men (n = 85)	Women (n = 164)	Race	Sex
Age, years	37.0 ± 4.2^¶^	36.7 ± 4.2^¶^	36.9 ± 3.8^¶^	35.5 ± 4.7^¶^	< 0.05^f^	NS
BMI, kg/m^2^	29.2 ± 5.8^¶^	28.3 ± 7.2^¶^	29.8 ± 7.1	32.9 ± 9.1	< 0.001^f^	< 0.01^b^
Waist/height	0.56 ± 0.08	0.53 ± 0.1^¶^	0.55 ± 0.09	0.59 ± 0.11	< 0.001^f^	< 0.05
Systolic blood pressure, mm Hg	118 ± 11	111 ± 11	129 ± 17	120 ± 16	< 0.001	< 0.001
Diastolic blood pressure, mm Hg	80 ± 8	75 ± 8	88 ± 13^‡^	80 ± 11	< 0.001	< 0.001
Fasting glucose, mg/dl	88 ± 24^¶^	84 ± 20^§^	91 ± 33	91 ± 35^§^	< 0.05^f^	NS
Fasting insulin, μU/ml^†^	12.8 ± 8.9	11.3 ± 8.3	12.2 ± 9.0	15.3 ± 12.1	< 0.001^f^	< 0.05
LDL cholesterol, mg/dl	130 ± 34^‡^	124 ± 33	124 ± 45	118 ± 33	NS	NS
Fasting triglycerides, mg/dl^†^	165 ± 129	118 ± 69	147 ± 126	88 ± 37	< 0.001^f^	< 0.001
HDL cholesterol, mg/dl	41 ± 12	51 ± 13^‡^	50 ± 13	51 ± 13	< 0.001^m^	< 0.001^w^
Total cholesterol/HDL cholesterol	5.0 ± 1.4	4.0 ± 1.1	4.3 ± 1.6^‡^	3.8 ± 1.2	< 0.001^m^	< 0.05
High-sensitivity C-reactive protein ≥ 2 mg/l	32.6%	47.3%	47.1%	58.1%	< 0.05	< 0.001^w^
Current smoker	34.7%	27.2%	53.6%	32.2%	< 0.01^m^	< 0.01^b^

Follow-up survey (2003-2005)						

Age, years	39.3 ± 4.2^¶^	39.1 ± 4.2^¶^	39.2 ± 3.8^¶^	37.9 ± 4.7^¶^	< 0.05^f^	NS
BMI, kg/m^2^	29.8 ± 6.2^¶^	28.8 ± 7.6^¶^	30.0 ± 7.5	33.6 ± 9.3	< 0.01^f^	NS
Waist/height	0.56 ± 0.08	0.54 ± 0.1^¶^	0.55 ± 0.1	0.59 ± 0.11	< 0.01^f^	NS
Systolic blood pressure, mm Hg	118 ± 12	111 ± 12	128 ± 17	121 ± 17	< 0.01	< 0.01^w^
Diastolic blood pressure, mm Hg	80 ± 9	75 ± 8	86 ± 14^‡^	81 ± 12	< 0.05	< 0.01^w^
Fasting glucose, mg/dl	94 ± 33^¶^	86 ± 17^§^	94 ± 25	96 ± 40^§^	< 0.01^f^	< 0.01^w^
LDL cholesterol, mg/dl	134 ± 39^‡^	125 ± 33	122 ± 40	119 ± 36	NS	NS
Fasting triglycerides, mg/dl^†^	174 ± 138	122 ± 69	142 ± 115	93 ± 51	< 0.01^f^	< 0.01
HDL cholesterol, mg/dl	42 ± 10	52 ± 13^‡^	51 ± 16	52 ± 13	< 0.01^m^	< 0.001^w^
Total cholesterol/HDL cholesterol	5.0 ± 1.4	3.9 ± 1.1	4.0 ± 1.4^‡^	4.2 ± 1.4	< 0.01^m^	< 0.001^w^
Current smoker	33.0%	28.7%	54.1%	32.1%	< 0.01^m^	< 0.01^b^

*Analysis of covariance (p value adjusted for age).

NS indicates p > 0.05; ^f^, females only; ^m^, males only; ^b^, blacks only; ^w^, whites only.

^† ^Mean values are presented and their Log transformed values were used to calculate p values.

^‡ ^p < 0.05 baseline survey vs follow-up survey.

^§ ^p < 0.01 baseline survey vs follow-up survey.

^¶ ^p < 0.001 baseline survey vs follow-up survey.

**Figure 1:**
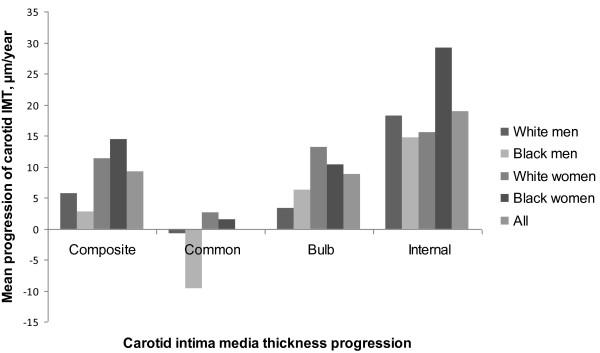
**Carotid IMT progression rates per year in black and white young adults**.

Carotid IMT measurements and progression rates were presented in Tables 2 and 3, respectively. At the baseline survey, significant race differences were noted in both sexes for common carotid artery IMT (whites < blacks); however, the race differences in composite carotid artery IMT (whites < blacks), and carotid bulb IMT (whites < blacks) were significant only in females. Compared with women, men showed higher values for composite, common, bulb, and internal carotid artery IMT. Significant race differences for composite carotid artery and carotid bulb IMT were no longer evident in the second survey. Composite carotid artery (all except black men), carotid bulb (white women), and internal carotid artery (all except black men) IMT showed significant progression between the two surveys (Table 2). Carotid IMT progression rates were as follows (Table 3): composite carotid artery = 9.2 ± 52 μm/y, common carotid artery = 0.0 ± 51 μm/y, carotid bulb = 8.8 ± 103 μm/y, and internal carotid artery = 18.9 ± 81 μm/y (Figure 1). Carotid artery IMT progression rates did not differ significantly among race-sex groups.

**Table 2:** Carotid artery IMT measurements in the baseline and follow-up surveys by race and sex

	White		Black		P value*	
	
Baseline survey (2001-2002)	Men (n = 266)	Women (n = 324)	Men (n = 85)	Women (n = 164)	Race	Sex
Composite CIMT, mm^†^	0.851 ± 0.175^‡^	0.760 ± 0.123^§^	0.897 ± 0.180	0.807 ± 0.161^¶^	< 0.01^f^	< 0.001
Common CIMT, mm^†^	0.785 ± 0.136	0.704 ± 0.102	0.841 ± 0.156	0.770 ± 0.133	< 0.01	< 0.001
Bulb CIMT, mm^†^	1.016 ± 0.311	0.907 ± 0.213^‡^	1.051 ± 0.352	0.943 ± 0.298	< 0.01^f^	< 0.01
Internal CIMT, mm^†^	0.742 ± 0.211^§^	0.674 ± 0.183^§^	0.786 ± 0.213	0.703 ± 0.170^¶^	NS	< 0.01

Follow-up survey (2003-2005)						

Composite CIMT, mm^†^	0.867 ± 0.174^‡^	0.790 ± 0.162^§^	0.892 ± 0.223	0.836 ± 0.164^¶^	NS	< 0.01^w^
Common CIMT, mm^†^	0.781 ± 0.143	0.711 ± 0.120	0.818 ± 0.171	0.775 ± 0.148	< 0.01^f^	< 0.001^w^
Bulb CIMT, mm^†^	1.032 ± 0.298	0.942 ± 0.280^‡^	1.058 ± 0.361	0.964 ± 0.256	NS	< 0.05^w^
Internal CIMT, mm^†^	0.793 ± 0.232^§^	0.711 ± 0.218^§^	0.820 ± 0.255	0.762 ± 0.198^¶^	NS	< 0.05^w^

*Analysis of covariance (p value adjusted for age).

NS indicates p > 0.05; ^f^, females only; ^w^, whites only.

^† ^Mean values are presented and their Log transformed values were used to calculate p values.

^‡ ^p < 0.05 baseline survey vs follow-up survey.

^§ ^p < 0.01 baseline survey vs follow-up survey.

^¶ ^p < 0.001 baseline survey vs follow-up survey.

**Table 3:** Carotid artery IMT progression rates

Site	Race and sex	N	Mean ΔCIMT μm/year	95% CI, μm/year	
				
				Lower	Upper
Composite CIMT*	All	695	9.2	5.3	13.0
	White men	231	5.7	-1.0	12.4
	Black men	70	2.8	-12.0	17.5
	White women	266	11.3	4.9	17.7
	Black women	128	14.5	6.5	22.3
Common CIMT*	All	799	0.0	-3.5	3.5
	White men	256	-0.8	-6.2	4.7
	Black men	84	-9.7	-24.3	4.9
	White women	302	2.6	-2.5	7.6
	Black women	157	1.5	-7.9	11.0
Bulb CIMT*	All	768	8.8	1.4	16.1
	White men	247	3.3	-9.6	16.1
	Black men	79	6.2	-20.1	32.5
	White women	297	13.2	1.4	25.1
	Black women	145	10.3	-5.3	25.8
Internal CIMT*	All	706	18.9	13.0	24.9
	White men	235	18.2	7.9	28.4
	Black men	71	14.7	-7.8	37.2
	White women	267	15.6	6.1	25.1
	Black women	133	29.2	15.9	42.5

*Analysis of variance p > 0.05 among race-sex groups.

Baseline CV risk correlates of the composite carotid artery IMT progression are presented in Table 4. Composite carotid artery IMT progression was adjusted for the baseline (2001-2002 survey) composite carotid artery IMT, which showed a significant association with an unstandardized difference of -0.296 ([95% CI between -0.353 and -0.240], p < 0.001). Based on the magnitude of adjusted difference in the total sample, increase in baseline age, systolic blood pressure, fasting glucose, prevalence of high sensitivity c-reactive protein ≥ 2 mg/l, LDL cholesterol, and prevalence of current smoking were, in that order, associated with the composite carotid artery IMT progression.

**Table 4:** Baseline independent cardiovascular risk correlates of the composite carotid artery IMT progression in young adults

	Univariate Associations (Unadjusted)		Multivariable Model Following Stepwise Selection (Adjusted for Baseline IMT)			
**Variables**	**Univariate** **Correlations**	**p value**	Unstandardized difference*	**95% CI**	Standardized difference*	**p value**

Age, years	0.199	< 0.001	0.006	0.004 - 0.008	0.208	< 0.001
Systolic blood pressure, mm Hg	0.165	< 0.001	0.002	0.001 - 0.002	0.171	< 0.001
Fasting glucose, mg/dl	0.129	< 0.001	0.001	0.000 - 0.001	0.126	< 0.01
hsCRP ≥ 2 mg/l	0.110	< 0.001	0.025	0.008 - 0.043	0.103	< 0.01
LDL cholesterol, mg/dl	0.085	< 0.01	0.000	0.000 - 0.001	0.088	< 0.05
Current smoker	0.091	< 0.05	0.021	0.003 - 0.039	0.084	< 0.05
Baseline IMT (microns)	(-0.369)	< 0.001	(-0.296)	(-0.353) - (-0.240)	(-0.405)	< 0.001
Gender (women vs men)	0.035	0.37	0.008	(-0.011) - 0.027	0.032	0.42
Race (black vs white)	(-0.025)	0.51	(-0.01)	(-0.031) - 0.011	-0.038	0.35
Diastolic blood pressure, mmHg	(-0.022)	0.57	0.000	(-0.002) - 0.001	-0.033	0.65
Waist-height ratio	(-0.002)	0.46	0.008	(-0.104) - 0.121	0.006	0.88
HDL cholesterol, mg/dl	0.021	0.6	0.000	(-0.001) - 0.001	0.014	0.72
Fasting triglycerides, mg/dl^†^	(-0.021)	0.59	(-0.004)	(-0.022) - 0.014	(-0.019)	0.65

Baseline adjusted stepwise linear regression model included age, gender, race, systolic and diastolic blood pressures, waist-height ratio, fasting glucose, serum low density lipoprotein cholesterol, high density lipoprotein cholesterol, fasting triglycerides^†^, high-sensitivity C-reactive protein ≥ 2 mg/l and smoking status.

*Unstandardized and standardized differences in mean composite carotid artery IMT progression.

^† ^Log transformed value was used in the model.

## 4. Discussion

In this large community population, we identified the association of baseline traditional CV risks and hsCRP to carotid artery IMT progression in young adults (24- to 43-year old) over a 2.4-year period. The major finding was that in addition to increase in baseline age, systolic blood pressure, glucose, LDL cholesterol and the prevalence of current smoking, increase in the prevalence of baseline hsCRP ≥ 2 mg/l were associated with the composite carotid artery IMT progression independent of other traditional CV risks. An earlier report from the Bogalusa Heart Study demonstrated that smoking was the most consistent predictor of carotid artery IMT progression in 336 young men and women, who were screened in both 1995-1996 and 2000-2001 surveys, during an average follow-up of 5.8 years [4]. The prior reported composite and common carotid artery IMT progression rates of 17 μm/y and 16 μm/y were much higher than the current composite and common of 9.2 μm/y and 0.0 μm/y in 839 young men and women. The rate of progression over a 2.4-year period is relatively short and may be responsible for the inability to detect a significant progression of IMT in the common carotid artery segment. Nevertheless, current report expands the findings of this earlier article, in which the association between carotid artery IMT and baseline CV risk factors were investigated in a smaller sample of population and relationship with hsCRP was not looked at.

A number of other large, prospective, and population-based cohort studies have investigated the association between hsCRP and carotid artery IMT progression. The INVADE (intervention project of cerebrovascular events and dementia in the community of Ebersberg) study [11] have found an independent association between baseline hsCRP and common carotid artery IMT progression after 2 years in elderly women but not in men. A considerable number of other epidemiologic studies have found no link between the increased baseline hsCRP levels with carotid artery IMT progression. The Cardiovascular Risk in Young Finns Study reported no association between hsCRP in childhood (3 to 18 years of age) with carotid artery IMT 21 years later [17]. The Rotterdam study [10] reported no relation between hsCRP categories and change in common carotid artery IMT during a mean follow-up period of 6.4 years. On the other hand, the same study also reported the relation of hsCRP and the progression of carotid atherosclerosis by the finding of independent and graded associations of hsCRP with the extent and progression of carotid plaques and ankle-brachial index in the elderly men and women ≥ 55 years of age [10]. Similarly, Carotid Atherosclerosis Progression Study [9] reported that hsCRP was not associated with carotid artery IMT progression after 3 years in middle-aged individuals. In our study, we have observed that hsCRP ≥ 2 mg/l was an independent risk factor for early composite carotid artery IMT progression for the whole study population of young adults.

The role of hsCRP as an acute phase reactant on the progression of early atherosclerosis has not been clarified in detail. High-sensitivity CRP levels might express the inflammatory activity of the atherosclerotic lesion, but hsCRP has been suggested to promote endothelial dysfunction and progression of atherosclerotic lesions in the early stages, and become more pronounced in advanced stages with an important role in plaque vulnerability, rapid progression of plaques, and thrombotic complications [18-20]. Considerable epidemiological evidence has linked increased hsCRP levels with incresed risk of CV events including myocardial infarction, stroke, death from CV causes, coronary revascularization, or a combination of these in middle aged and elderly subjects [9,21-28]. A number of these have suggested this association is independent of traditional CV risk factors [21-27], although others have found it attenuated when risk factors are controlled for [9,28]. A number of potential mechanisms by which CRP may play a causal role in atherosclerosis and CV disease have been implicated, including recruitment of monocytes to the atherosclerotic lesion [29], intimal growth [30], and endothelial dysfunction [19,31].

As might be expected, this study identified baseline predictors of longitudinal carotid artery IMT progression, it is an association study and it is subject to selection bias. CRP is produced in the liver and its concentration is increased by infections that show no relationship to atherosclerosis, as well as by malignancy. Intima-media thickening reflects early atherosclerotic change as well as vessel remodeling. The number of subjects with plaques was insufficient to investigate associations with more advanced atherosclerosis in our population. Restricting measurements of IMT to the common carotid artery segment has been justified in other studies by greater reproducibility of measurements from this site and the difficulty in obtaining measurements from the bifurcation or the internal carotid artery in some populations. In the present study, 87% of all subjects had valid IMT measurements from all segments. Protocols that involve additional segments have several advantages [5]. First, progression is more prominent in the bifurcation and the internal carotid artery. Thus, including these sites may provide the most sensitive and statistically powerful assessment of atherosclerosis progression. Yet, aggregating data across segments may provide measures that are stable and less sensitive to measurement error.

Some methodologic limitations must be taken into account in the interpretation of results. The rate of progression over a 2.4-year period is relatively short and combined with measurement errors, which could be related in part to two different ultrasound machine recordings between surveys, may be responsible for the inability to detect a significant progression of IMT in the common carotid artery segment. Nevertheless, we have identified associations between traditional CV risk factors, hsCRP and composite carotid artery IMT in cross sectional studies in this population. Longer-term follow-up should increase the power to detect other associations and reveal race and gender differences.

## Conclusion

In a prospective, population-based study, we observed independent categorical association of increased hsCRP and multiple conventional risk factors including smoking with carotid artery IMT progression in young adults. This study underlines the importance of assesssing CV risk factor profiles and hsCRP level in young adults. These observations demonstrate that risk factors are associated with the progression of subclinical atherosclerosis even in young individuals who are at low risk for cardiac events. These observations reflect on the CV risk burden over time by risk factors including inflammation and smoking.

## Abbreviations

hsCRP: high sensitivity c-reactive protein; CV: cardiovascular; IMT: intima-media thickness; LDL: low-density lipoprotein cholesterol; BMI: body mass index; HDL: high-density lipoprotein cholesterol; INVADE: intervention project of cerebrovascular events and dementia in the community of; Ebersberg; CIMT: carotid artery intima-media thickness.

## Competing interests

The authors declare that they have no competing interests.

## Authors' contributions

All authors read and approved the final manuscript. AT: Participated in its design and coordination and drafted the manuscript. RK: Involved in drafting the manuscript and revising it critically for important intellectual content. DT: Involved in drafting the manuscript and revising it critically for important intellectual content. WC: Have made substantial contributions to conception and design, acquisition of data, analysis and interpretation of data, and have given final approval of the version to be published. SS: Have made substantial contributions to conception and design, acquisition of data, analysis and interpretation of data, and have given final approval of the version to be published. JHX Have made substantial contributions to conception and design, acquisition of data, analysis and interpretation of data, and have given final approval of the version to be published. AA: Involved in drafting the manuscript and revising it critically for important intellectual content. GSB: Have made substantial contributions to conception and design, acquisition of data, analysis and interpretation of data, and have given final approval of the version to be published.

## Pre-publication history

The pre-publication history for this paper can be accessed here:

http://www.biomedcentral.com/1471-2261/11/78/prepub
